# Individualized OnabotulinumtoxinA Treatment for Upper Limb Spasticity Resulted in High Clinician‐ and Patient‐Reported Satisfaction: Long‐Term Observational Results from the ASPIRE Study

**DOI:** 10.1002/pmrj.12328

**Published:** 2020-02-27

**Authors:** Gerard E. Francisco, Wolfgang H. Jost, Ganesh Bavikatte, Daniel S. Bandari, Simon F.T. Tang, Michael C. Munin, Joan Largent, Aubrey M. Adams, Aleksej Zuzek, Alberto Esquenazi

**Affiliations:** ^1^ The University of Texas Health Science Center McGovern Medical School and TIRR Memorial Hermann Houston TX; ^2^ Department of Neurology University of Freiburg Freiburg im Breisgau Germany; ^3^ The Walton Centre Liverpool UK; ^4^ Multiple Sclerosis Center of California & Research Group Newport Beach CA; ^5^ Department of Physical Medicine and Rehabilitation Lotung Poh‐Ai Hospital Yilan Taiwan; ^6^ Department of Physical Medicine and Rehabilitation University of Pittsburgh School of Medicine Pittsburgh PA; ^7^ IQVIA Real‐World Evidence Solutions Cambridge MA; ^8^ Allergan plc Irvine CA; ^9^ MossRehab Gait and Motion Analysis Laboratory Elkins Park PA

## Abstract

**Introduction:**

OnabotulinumtoxinA treatment for spasticity is dependent on numerous factors and varies according to selected treatment goals.

**Objective:**

To examine real‐world onabotulinumtoxinA treatment utilization and effectiveness in patients with upper limb spasticity over 2 years from the Adult Spasticity International Registry (ASPIRE) study.

**Design:**

Multicenter, prospective, observational registry (NCT01930786).

**Setting:**

Fifty‐four international clinical sites in North America, Europe, and Asia.

**Patients:**

Adults (naïve or non‐naïve to botulinum toxins for spasticity) with upper limb focal spasticity related to upper motor neuron syndrome across multiple etiologies.

**Interventions:**

OnabotulinumtoxinA administered at clinician's discretion.

**Main Outcome Measures:**

OnabotulinumtoxinA utilization, clinician and patient satisfaction.

**Results:**

Four hundred eighty‐four patients received ≥1 treatment of onabotulinumtoxinA for upper limb spasticity. Patients were on average 55.1 years old, 50.8% male, predominantly Caucasian (72.3%), and 38.6% were naïve to botulinum toxins. Stroke was the most frequently reported underlying etiology (74.0%). Most patients (81.2%) had moderate to severe spasticity at baseline. The most commonly treated upper limb clinical presentation was clenched fist (79.1% of patients). Across all presentations, onabotulinumtoxinA doses ranged between 5‐600U. Electromyography (EMG) was most often utilized to localize muscles (≥57.0% of treatment sessions). Clinicians (92.9% of treatment sessions) and patients (85.7%) reported being extremely satisfied/satisfied that treatment helped manage spasticity, and clinicians (98.6%) and patients (92.2%) would definitely/probably continue onabotulinumtoxinA treatment. One hundred seventy‐nine patients (37.0%) reported 563 adverse events (AEs); 15 AEs in 14 patients (2.9%) were considered treatment related. Sixty‐nine patients (14.3%) reported 137 serious AEs; 3 serious AEs in 2 patients (0.4%) were considered treatment related. No new safety signals were identified.

**Conclusions:**

ASPIRE captured the real‐world individualized nature of onabotulinumtoxinA utilization for upper limb spasticity over 2 years, with consistently high clinician‐ and patient‐reported satisfaction. Data in this primary analysis will guide clinical use of onabotulinumtoxinA, as well as provide insights to improve educational programs on spasticity management.

## Introduction

Spasticity can be defined as disordered sensorimotor control, resulting from an upper motor neuron lesion, presenting as intermittent or sustained involuntary activation of muscles.^1^, ^2^ Several central nervous system disorders are associated with spasticity, including stroke, cerebral palsy, multiple sclerosis, spinal cord injury, and traumatic brain injury.^3^, ^4^, ^5^ Upper limb spasticity affects the shoulder, elbow, wrist, and finger flexors, resulting in abnormal postures.^6^, ^7^ Upper limb spasticity affects both active and passive function, which can interfere with limb dexterity and mobility, activities of daily living, and lead to limb pain.^4^, ^7^, ^8^, ^9^ Due to its negative impact on emotional and physical function, spasticity can result in lower quality of life and higher caregiver and economic burden.^10^, ^11^, ^12^, ^13^, ^14^

Clinical approaches to treat spasticity should be tailored to meet the needs and realistic goals of each patient, and often aim to improve quality of life and prevent secondary complications.^5^ Several treatment options to manage spasticity are available (reviewed in several studies^3^, ^4^, ^5^, ^8^, ^15^, ^16^), including oral medications, botulinum toxins, intrathecal baclofen, and procedures/surgeries, with combination treatments often recommended.^17^, ^18^ As many daily activities and occupations require high manual dexterity, treatments that improve occupational therapy outcomes are especially important for patients with upper limb spasticity. OnabotulinumtoxinA (Botox, Allergan plc, Dublin, Ireland) is approved for use in upper and lower limb spasticity in the United States and worldwide.^19^ OnabotulinumtoxinA is a focal neuromodulator that blocks acetylcholine release at neuromuscular junctions, leading to muscle relaxation.^19^, ^20^

There is a large body of evidence demonstrating the efficacy and safety of onabotulinumtoxinA for the treatment of upper limb spasticity in controlled trials (eg,^21^, ^22^, ^23^, ^24^, ^25^, ^26^, ^27^, ^28^ and reviewed in^8^, ^29^), with recommended use in clinical practice.^8^, ^16^, ^30^, ^31^ However, published onabotulinumtoxinA treatment utilization data in real‐world settings are limited but are necessary to help guide clinicians on common treatment practices to better meet the needs of patients. The Adult SPasticity International REgistry (ASPIRE) study was developed to describe the clinical characteristics of patients being treated with onabotulinumtoxinA for spasticity across multiple etiologies and geographical regions over 2 years.^32^ The main objectives of the ASPIRE study were to examine the patterns of onabotulinumtoxinA utilization and assess the effectiveness of onabotulinumtoxinA treatment for spasticity. This article focuses on patients from ASPIRE treated for upper limb spasticity, defined as any enrolled patient who received at least one treatment of onabotulinumtoxinA to the upper limb during the study period.

## Methods

A complete description of the study methods have been published previously^32^ and are described in brief here.

### *Study Design and Setting*

ASPIRE is an international, multicenter, prospective, observational registry (NCT01930786). Data were collected by 74 treating clinicians at 54 international sites in the United States, Spain, Germany, the United Kingdom, France, Italy, and Taiwan. OnabotulinumtoxinA treatments were administered at the clinician's discretion in accordance with usual clinical practices and country‐specific regulations. For most patients, re‐treatment is expected to occur approximately every 12 weeks according to the package inserts.^19^, ^33^ Financial support was not provided by the sponsor for any treatment/treatment related costs. ASPIRE included a 96‐week study period, followed by a 12‐week follow‐up period (108 weeks total). For study completion, patients had to meet the following criteria: (1) did not discontinue within the 96‐week study period; (2) were not lost to follow‐up; and (3) completed the final assessment form. ASPIRE was conducted in accordance with all relevant regulatory requirements, including the Declaration of Helsinki and the Guidelines for Good Pharmacoepidemiology Practices (International Society for Pharmacoepidemiology [IPSE]).

### *Participants*

Adult patients (≥18 years of age, men and women, naïve or non‐naïve to botulinum toxin[s] for spasticity) were treated with onabotulinumtoxinA for focal spasticity related to upper motor neuron syndrome during routine clinical practice. Inclusion/exclusion criteria are detailed in Francisco et al.^32^ All patients were required to provide written informed consent. Institutional review board approval was granted at each study site.

### *Outcomes and Data Sources*

Patient demographics and clinical characteristic data, including assessment of the patient's severity of spasticity using the Modified Modified Ashworth Scale (MMAS^34^), were collected at baseline. The primary objectives of ASPIRE were to (1) determine the patterns of utilization of onabotulinumtoxinA treatment for spasticity in clinical practice; and (2) quantify the effectiveness of onabotulinumtoxinA for the treatment of spasticity in clinical practice using clinician‐ and patient‐reported satisfaction. OnabotulinumtoxinA utilization data were captured at each treatment session. Clinician satisfaction was collected at each subsequent treatment session and patient satisfaction was collected 5 ± 1 weeks post‐treatment via phone or web.

The secondary objectives relevant to this analysis include (1) patient‐reported outcome (PRO) data to evaluate the impacts of spasticity on quality of life, physical function, activities of daily life, and pain; and (2) estimation of the incidence of adverse events (AEs). PRO data included the Disability Assessment Scale (DAS^35^), which was assessed by the clinician at treatment session 1 and at each subsequent treatment session, and the Numeric Pain Rating Scale (NPRS^36^, ^37^), which was self‐reported by patients at baseline and 5 ± 1 weeks post‐treatment via phone or web. AE data were captured throughout the study (108 weeks total) and were summarized using the Medical Dictionary for Regulatory Activities (MedDRA) version 20.0 by system organ class and preferred term. Relationship to treatment and evaluation of potential distant spread of toxin were adjudicated by a panel of safety clinicians. For additional details on the assessment scales utilized in ASPIRE, as well as a complete list of data collected, refer to Francisco et al.^32^

### *Control for Bias*

ASPIRE was designed for high generalizability to clinical practice. To minimize selection bias, broad eligibility criteria were applied to capture real‐world onabotulinumtoxinA utilization for spasticity, including multiple etiologies and geographical regions, as well as patients naïve or non‐naïve to botulinum toxins for spasticity. However, inclusion of non‐naïve patients may have introduced a selection bias for patients in which onabotulinumtoxinA was tolerable and effective. To minimize this bias, ASPIRE aimed to enroll approximately one‐third of patients that were naïve to botulinum toxins for spasticity in addition to non‐naïve patients. To minimize information bias, case report forms were carefully designed, and training provided to site staff, with assessments performed by the contract research organization to ensure data quality.

### *Study Size, Statistical Methods, and Analysis Populations*

No formal sample size/statistical power calculations were performed, as analyses of the primary study objectives were descriptive and did not test specific hypotheses. Data collected beyond 108 weeks were not included. Observed data are shown; no imputation of missing values was performed. Statistical significance was determined using ordinal logistic regression for DAS and paired *t*‐tests with Bonferroni correction for NPRS. Statistics were performed using SAS version 9.2 or higher (SAS Institute, Cary, NC).

The total analysis population includes all enrolled patients who received at least one dose of onabotulinumtoxinA during the study and was subdivided into the upper limb spasticity population and the lower limb spasticity population for publication. For this analysis of the upper limb spasticity population, all enrolled patients (naïve and non‐naïve to botulinum toxins for spasticity) who received at least one treatment of onabotulinumtoxinA to the upper limb during the study period were included. Data were collected by clinical presentation; patients could have been treated for ≥1 presentation. Importantly, patients in the upper limb spasticity population may have also received treatment to the lower limb during the study; however, only upper limb data are described here.

## Results

### *Patient Disposition*

ASPIRE (study dates: 16 Oct 2013 to 9 Oct 2017) enrolled 744 patients. For the total analysis population, 14 patients were excluded (*N* = 14/744, 1.9%; Figure S1) and 730 patients were included (*N* = 730/744, 98.1%). Over the 2‐year study, 484 patients received at least one treatment of onabotulinumtoxinA to the upper limb during at least one treatment session, hereafter referred to as the upper limb (spasticity) population. Patients who received treatment to the lower limb only (*N* = 246) were excluded from this analysis. Of those in the upper limb population (*N* = 484), 285 patients (58.9%) completed the study and 199 patients (41.1%) discontinued the study. Of those that discontinued, 121 patients (60.8%) withdrew consent, 63 patients (31.7%) failed to complete the final assessment form, and 15 patients (7.5%) were lost to follow‐up. The most common patient‐reported reasons for withdrawal of consent were treatment ineffective (*N* = 45/484, 9.3%) and difficulty paying for onabotulinumtoxinA treatment (*N* = 25/484, 5.2%). A full list of reasons for discontinuation is provided in Table S1.

### *Demographics and Clinical Characteristics*

**Figure 1:**
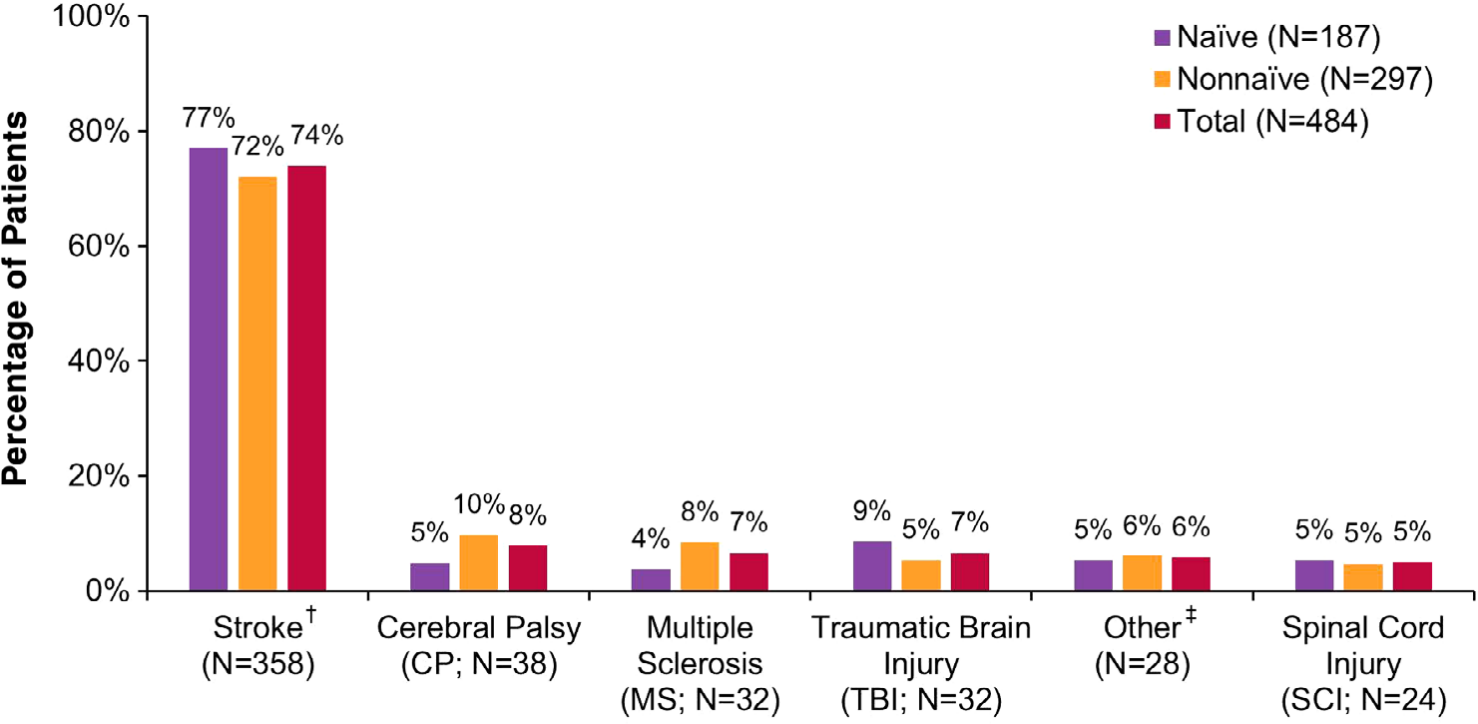
Primary etiology of spasticity at baseline in the upper limb spasticity population. For each population shown (ie, naïve, non‐naïve, and total), percentages sum to 100% across etiologies. Etiologies were not mutually exclusive, as more than one response was allowed per patient. ^†^Stroke includes ischemic, hemorrhagic, and embolic stroke. ^‡^Other includes hereditary spastic paraparesis, stroke during aneurysm clipping, chiari malformation, and hydrocephalus. CP, cerebral palsy; MS, multiple sclerosis; *N*, number of patients; TBI, traumatic brain injury; SCI, spinal cord injury.

**Figure 2:**
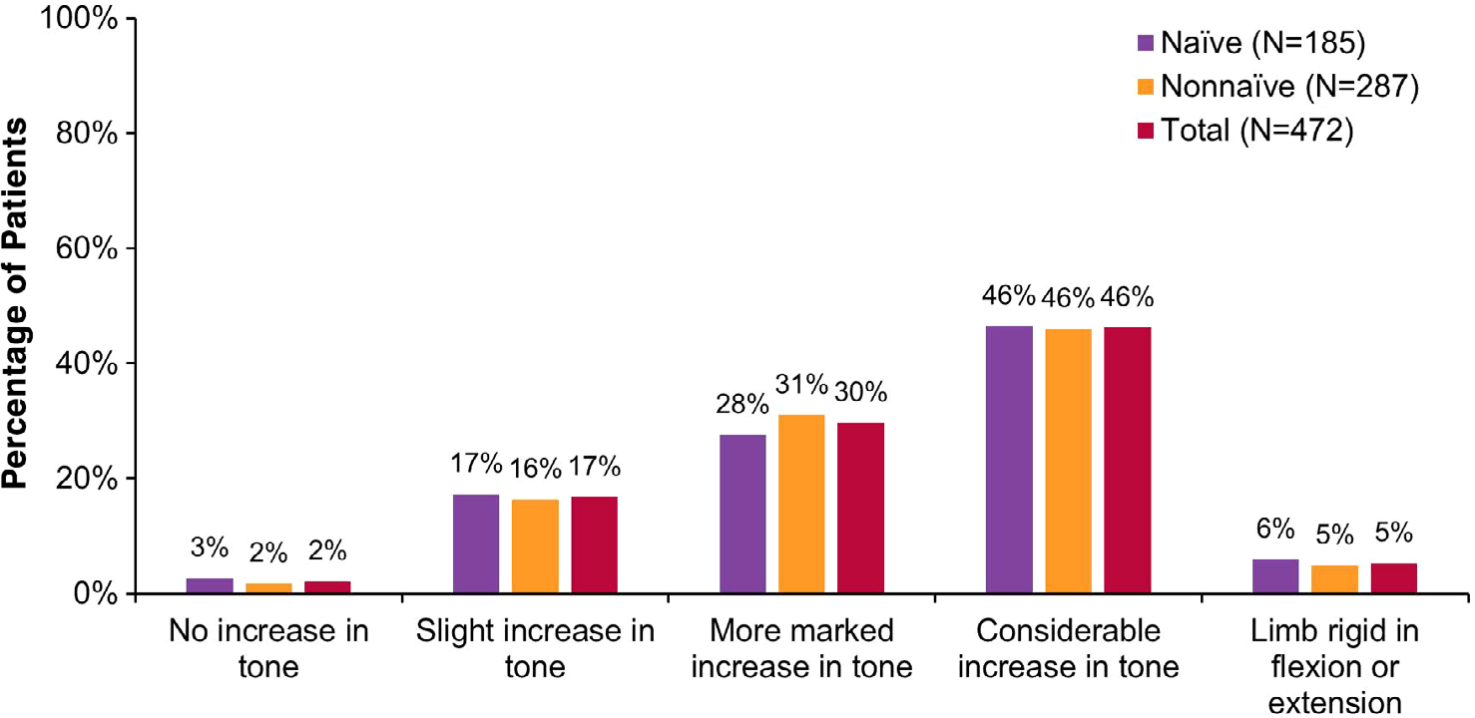
Severity of spasticity at baseline in the upper limb spasticity population. Severity of spasticity was determined at baseline for each clinical presentation using the Modified Modified Ashworth Scale (MMAS^34^). Data shown within the figure represents the mean MMAS score at baseline across all upper limb clinical presentations. For each population shown (ie, naïve, non‐naïve, and total), percentages sum to 100% across MMAS categories. MMAS data are missing for 12 patients (2 naïve, 10 non‐naïve). *N*, number of patients.

At baseline, patients were on average 55 years old, predominantly Caucasian (*N* = 350/484, 72.3%), evenly distributed by gender (men: *N* = 246/484, 50.8%), and most patients (*N* = 297/484, 61.4%) were non‐naïve to botulinum toxins for spasticity (Table 1). Patient demographics for the upper limb population were similar to the total population, as described previously.^32^ Stroke was the most commonly reported underlying etiology of spasticity (*N* = 358/484, 74.0%; Figure 1). The majority of patients (*N* = 358/472, 75.9%) had either more marked or considerable increase in tone, as evaluated by the MMAS (Figure 2).^34^ For additional baseline data, including oral medications, treatment modalities, SF‐12 domain scores, as well as caregiver and clinician demographics, refer to Francisco et al.^32^

**Table 1:** Baseline patient demographics for the upper limb spasticity population

	(*N* = 484)
Age (y)	
Mean (SD)	55.1 (15.3)
Median	57.0
Min, Max	19.2, 93.2
Gender, *N* (%)	
Female	238 (49.2)
Male	246 (50.8)
Race, *N* (%)	
Caucasian	350 (72.3)
Black/African/Caribbean	69 (14.3)
Asian	39 (8.1)
Latino/Hispanic	16 (3.3)
Middle Eastern/Arab	3 (0.6)
American Indian/Alaska Native	1 (0.2)
Other	2 (0.4)
Data Not Available	4 (0.8)
BMI (kg/m^2^), *N*	402
Mean (SD)	26.7 (5.5)
Median	25.8
Min, Max	14.9, 56.8
Naïve to botulinum toxin for spasticity, *N* (%)	
Yes	187 (38.6)

BMI = body mass index; Max = maximum; Min = minimum; *N =* number of patients.

### *OnabotulinumtoxinA Treatment Utilization*

**Figure 3:**
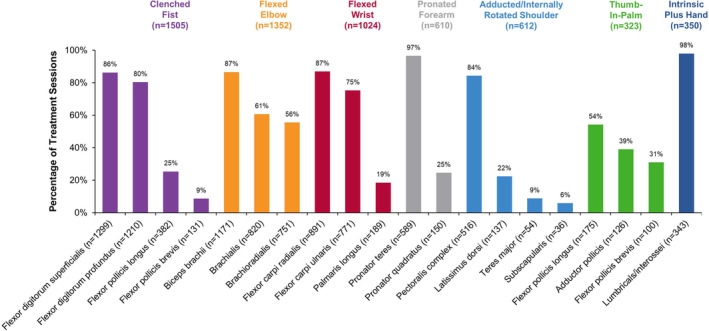
Muscles injected with onabotulinumtoxinA for the treatment of upper limb spasticity, stratified by clinical presentation. Clinical presentations are listed in order of number of patients treated: highest to lowest. Upper limb spasticity presentations, and muscles within each presentation, are not mutually exclusive, and therefore, categories may sum to >100%. Data for “other” clinical presentations and “other” muscles not predefined within the case report form, including for nonspasticity indications, are not shown. *n*, number of treatment sessions per clinical presentation or muscle injected.

Over 2 years, onabotulinumtoxinA was administered in 1974 treatment sessions to the upper limb spasticity population (*N* = 484). The mean (SD) treatment interval across all treatment sessions was 17.1 (7.1) weeks. The most common upper limb clinical presentation treated in ASPIRE (as determined by number of patients) was clenched fist, followed by flexed elbow, flexed wrist, pronated forearm, adducted/internally rotated shoulder, thumb‐in‐palm, and intrinsic plus hand (refer to Simpson et al^31^ and Mayer and Esquenazi^38^ for presentation descriptions). Data in Table 2 and Figure 3 demonstrate the real‐world diversity of onabotulinumtoxinA treatment for each clinical presentation individually, including dose and dilution, needle length, number of injections, treatment side, injection localization methods, and muscles targeted, with findings of interest highlighted here.

**Table 2:** OnabotulinumtoxinA treatment utilization in patients treated for upper limb spasticity (*N* = 484), stratified by clinical presentation*

	Clenched	Flexed	Flexed	Pronated	Adducted	Thumb‐	Intrinsic
	Fist	Elbow	Wrist	Forearm	Shoulder	In‐Palm	Plus Hand
Patients, *N* (%)	383 (79.1)	367 (75.8)	284 (58.7)	191 (39.5)	185 (38.2)	147 (30.4)	119 (24.6)
Treatment Sessions, *n*	1505	1352	1024	610	612	323	350
Dose (U)							
Mean (SD)	106 (70)	116 (74)	80 (59)	46 (29)	91 (57)	35 (30)	45 (22)
Mode	100	100	100	50	100	20	50
Min, Max	10, 525	15, 600	10, 500	10, 250	12, 450	5, 300	5, 125
Dilution (U/mL),† *n* (%)							
< 25	32 (2.2)	51 (3.8)	28 (2.7)	17 (2.8)	18 (2.9)	1 (0.3)	0 (0.0)
25	112 (7.4)	106 (7.8)	51 (5.0)	49 (8.0)	82 (13.4)	16 (5.0)	13 (3.7)
50	664 (44.1)	609 (45.0)	451 (44.0)	281 (46.1)	231 (37.7)	125 (38.7)	192 (54.9)
100	572 (38.0)	472 (34.9)	412 (40.2)	227 (37.2)	248 (40.5)	161 (49.8)	137 (39.1)
Other	134 (8.9)	119 (8.8)	86 (8.4)	37 (6.1)	33 (5.4)	21 (6.5)	8 (2.3)
Needle Length (mm),† *n* (%)							
10	128 (8.5)	103 (7.6)	80 (7.8)	39 (6.4)	14 (2.3)	30 (9.3)	26 (7.4)
37	828 (55.0)	760 (56.2)	562 (54.9)	341 (55.9)	333 (54.4)	186 (57.6)	190 (54.3)
50	245 (16.3)	197 (14.6)	161 (15.7)	90 (14.8)	114 (18.6)	54 (16.7)	83 (23.7)
75	9 (0.6)	6 (0.4)	10 (1.0)	0 (0.0)	2 (0.3)	3 (0.9)	1 (0.3)
Other	317 (21.1)	297 (22.0)	214 (20.9)	140 (23.0)	152 (24.8)	53 (16.4)	52 (14.9)
Injections,† n (%)							
1	172 (11.4)	191 (14.1)	207 (20.2)	385 (63.1)	205 (33.5)	205 (63.5)	42 (12.0)
2	503 (33.4)	290 (21.4)	524 (51.2)	201 (33.0)	206 (33.7)	80 (24.8)	51 (14.6)
3	283 (18.8)	275 (20.3)	119 (11.6)	13 (2.1)	91 (14.9)	21 (6.5)	112 (32.0)
4	241 (16.0)	312 (23.1)	115 (11.2)	8 (1.3)	53 (8.7)	15 (4.6)	128 (36.6)
5	98 (6.5)	69 (5.1)	13 (1.3)	3 (0.5)	29 (4.7)	0 (0.0)	8 (2.3)
≥ 6	208 (13.8)	215 (15.9)	46 (4.5)	0 (0.0)	28 (4.6)	2 (0.6)	9 (2.5)
Treatment Side,† *n* (%)							
Right	664 (44.1)	607 (44.9)	512 (50.0)	256 (42.0)	253 (41.3)	136 (42.1)	120 (34.3)
Left	792 (52.6)	713 (52.7)	498 (48.6)	352 (57.7)	347 (56.7)	184 (57.0)	224 (64.0)
Both	49 (3.3)	32 (2.4)	14 (1.4)	2 (0.3)	12 (2.0)	3 (0.9)	6 (1.7)
Localization Method,‡ *n* (%)							
Anatomical	531 (35.3)	432 (32.0)	310 (30.3)	129 (21.1)	203 (33.2)	85 (26.3)	115 (32.9)
E‐stim	279 (18.5)	168 (12.4)	154 (15.0)	90 (14.8)	49 (8.0)	48 (14.9)	36 (10.3)
EMG	861 (57.2)	771 (57.0)	595 (58.1)	397 (65.1)	374 (61.1)	185 (57.3)	208 (59.4)
Ultrasound	395 (26.2)	303 (22.4)	261 (25.5)	156 (25.6)	189 (30.9)	89 (27.6)	97 (27.7)

EMG = electromyography; E‐stim = electrical stimulation; Max = maximum; mL = milliliter; mm = millimeter; Min = minimum; *N* = number of patients; *n* = number of treatment sessions; U = units of onabotulinumtoxinA.

* Upper limb spasticity presentations and muscles are not mutually exclusive, and therefore, do not add up to 100%.

† Data represent the sum, per clinical presentation, across all treatment sessions in the 2‐year study. Dilution and needle length categories are not mutually exclusive.

‡ Injection localization methods were not mutually exclusive and may have been influenced by availability of equipment at the site. “Anatomical” localization refers to palpation. Data represent the sum, per clinical presentation, across all treatment sessions in the 2‐year study.

#### Clenched Fist

In total, 383 patients received onabotulinumtoxinA for clenched fist in 1505 treatment sessions (Table 2). The most common dose (mode) of onabotulinumtoxinA per treatment session for clenched fist was 100 U. Of the available injection guidance techniques, clinicians frequently located the site(s) for injection using electromyography (EMG; *n* = 861/1505, 57.2%). For most treatment sessions, onabotulinumtoxinA was injected into the flexor digitorum superficialis (*n* = 1299/1505, 86.3%) and the flexor digitorum profundus (*n* = 1210/1505, 80.4%; Figure 3).

#### Flexed Elbow

In total, 367 patients received onabotulinumtoxinA for flexed elbow in 1352 treatment sessions (Table 2). The most common dose of onabotulinumtoxinA per treatment session for flexed elbow was 100 U. Clinicians most commonly located the site(s) for injection using EMG (*n* = 771/1352, 57.0%). For most treatment sessions, onabotulinumtoxinA was injected into the biceps brachii (*n* = 1171/1352, 86.6%), followed by brachialis (*n* = 820/1352, 60.7%) and brachioradialis (*n* = 751/1352, 55.5%; Figure 3).

#### Flexed Wrist

In total, 284 patients received onabotulinumtoxinA for flexed wrist in 1024 treatment sessions (Table 2). The most common dose of onabotulinumtoxinA per treatment session for flexed wrist was 100 U. Clinicians most commonly located the site(s) for injection using EMG (*n* = 595/1024, 58.1%). For most treatment sessions, onabotulinumtoxinA was injected into the flexor carpi radialis (*n* = 891/1024, 87.0%) and the flexor carpi ulnaris (*n* = 771/1024, 75.3%; Figure 3).

#### Pronated Forearm

In total, 191 patients received onabotulinumtoxinA for pronated forearm in 610 treatment sessions (Table 2). The most common dose of onabotulinumtoxinA per treatment session for pronated forearm was 50 U. Clinicians most commonly located the site(s) for injection using EMG (*n* = 397/610, 65.1%). At nearly all treatment sessions, onabotulinumtoxinA was injected into the pronator teres (*n* = 589/610, 96.6%; Figure 3).

#### Adducted/Internally Rotated Shoulder

In total, 185 patients received onabotulinumtoxinA for adducted/internally rotated shoulder in 612 treatment sessions (Table 2). The most common dose of onabotulinumtoxinA per treatment session for adducted/internally rotated shoulder was 100 U. Clinicians most commonly located the site(s) for injection using EMG (*n* = 374/612, 61.1%). For most treatment sessions, onabotulinumtoxinA was injected into the pectoralis complex (*n* = 516/612, 84.3%; Figure 3).

#### Thumb‐in‐Palm

In total, 147 patients received onabotulinumtoxinA for thumb‐in‐palm in 323 treatment sessions (Table 2). The most common dose of onabotulinumtoxinA per treatment session for thumb‐in‐palm was 20 U. Clinicians most commonly located the site(s) for injection using EMG (*n* = 185/323, 57.3%). For approximately half of treatment sessions, onabotulinumtoxinA was injected into the flexor pollicis longus (*n* = 175/323, 54.2%; Figure 3).

#### Intrinsic Plus Hand

In total, 119 patients received onabotulinumtoxinA for intrinsic plus hand in 350 treatment sessions (Table 3). The most common dose of onabotulinumtoxinA per treatment session for intrinsic plus hand was 50 U. Clinicians most commonly located the site(s) for injection using EMG (*n* = 208/350, 59.4%). For nearly all treatment sessions, onabotulinumtoxinA was injected into the lumbricals/interossei (*n* = 343/350, 98.0%; Figure 3).

#### Adjustments to Muscles Targeted and Dose of OnabotulinumtoxinA

**Figure 4:**
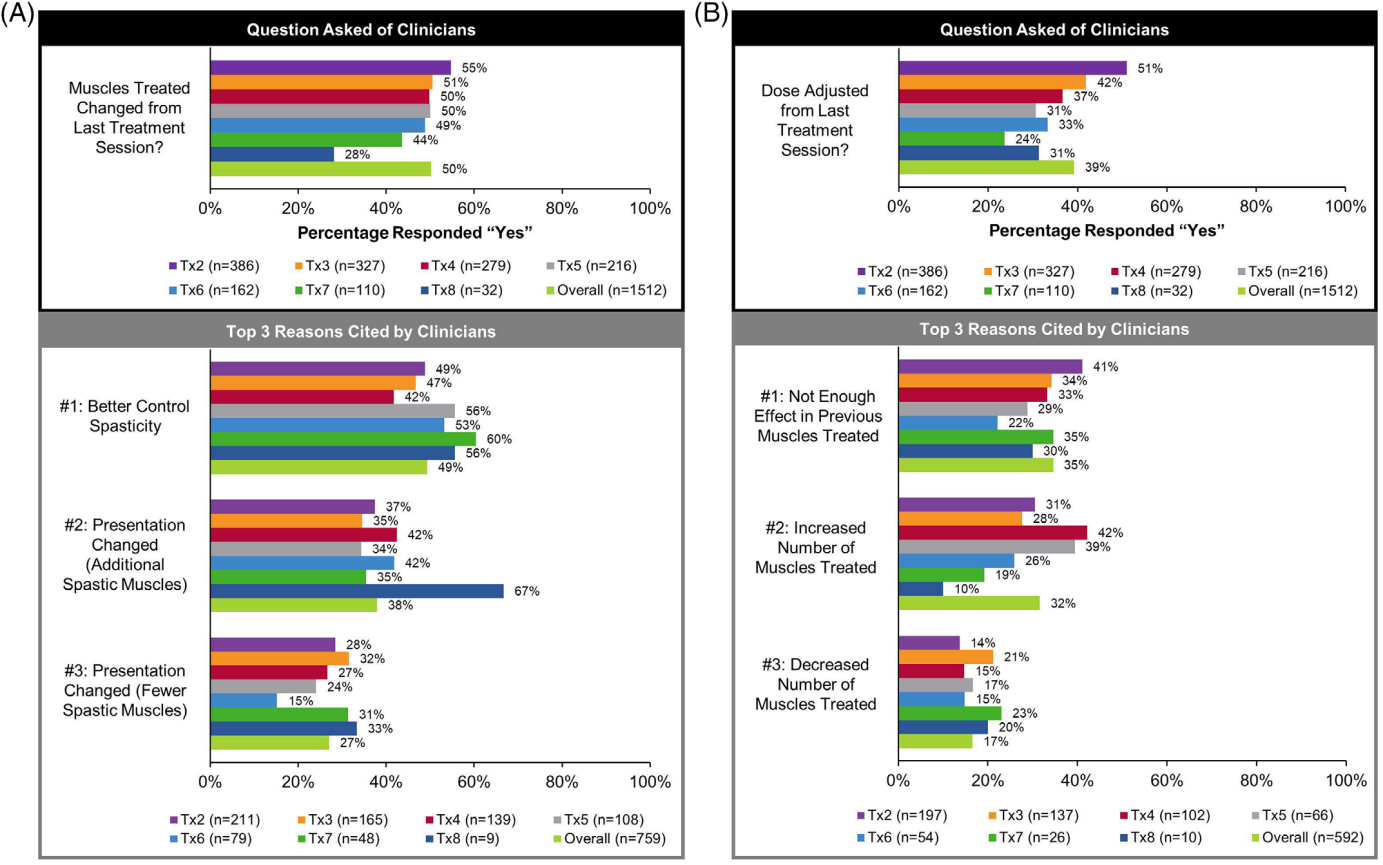
Adjustments to muscles targeted and dose of onabotulinumtoxinA at time of re‐treatment in the upper limb spasticity population. At the time of re‐treatment, clinicians were asked (A) if the muscles treated changed from the last treatment session and (B) if the dose was adjusted from the last treatment session (shown in black box). Of those clinicians that responded “yes” to the questions above, the top 3 reasons cited for this decision (excluding “other”) are provided in rank order (shown in gray box), where more than one reason was allowed. *n*, number of treatment sessions; Tx, treatment session.

At re‐treatment, clinicians were asked (1) if the muscles treated changed and (2) if the dose was adjusted, from the last treatment session (Figure 4). Clinicians adjusted the muscles treated in half of treatment sessions (overall: *n* = 759/1512, 50.2%; Figure 4A), with the top reason being to better control spasticity (overall: *n* = 374/759, 49.3%). For over a third of treatment sessions (overall: *n* = 592/1512, 39.2%), clinicians adjusted the dose of onabotulinumtoxinA administered (Figure 4B), with the top reason being not enough effect in previous muscles treated (overall: *n* = 205/592, 34.6%).

### *Effectiveness*

#### Disability Assessment Scale (DAS)

Compared to treatment session 1, onabotulinumtoxinA significantly improved DAS scores at subsequent treatments for all subscales, indicative of decreased functional impairment over time (all comparisons, *P* < .0001; Table 3).

**Table 3:** Disability Assessment Scale (DAS) in the upper limb spasticity population*

	Tx1	Tx2	Tx3	Tx4	Tx5	Tx6	Tx7	Tx8
	(*N* = 483)‡	(*N* = 410)	(*N* = 348)	(*N* = 295)	(*N* = 226)	(*N* = 169)	(*N* = 112)	(*N* = 33)
Dressing, *N* (%)								
0 ‐ No disability	49 (10.1)	57 (13.9)	42 (12.1)	41 (13.9)	23 (10.2)	26 (15.4)	11 (9.8)	4 (12.1)
1 ‐ Mild disability	129 (26.7)	150 (36.7)	141 (40.5)	117 (39.7)	102 (45.1)	74 (43.8)	49 (43.8)	15 (45.5)
2 ‐ Moderate disability	205 (42.4)	142 (34.7)	115 (33.0)	106 (35.9)	74 (32.7)	49 (29.0)	38 (33.9)	10 (30.3)
3 ‐ Severe disability	100 (20.7)	60 (14.7)	50 (14.4)	31 (10.5)	27 (11.9)	20 (11.8)	14 (12.5)	4 (12.1)
OR (95% CI)		2.3 (1.7, 3.1)	2.4 (1.8, 3.3)	3.0 (2.2, 4.2)	2.9 (2.0, 4.2)	3.4 (2.2, 5.1)	2.4 (1.5, 3.9)	4.2 (1.9, 9.6)
*F* Value: 9.5; *P* < .0001†								
Hygiene, *N* (%)								
0 ‐ No disability	97 (20.1)	105 (25.6)	90 (25.9)	82 (27.8)	63 (27.9)	61 (36.1)	36 (32.1)	10 (30.3)
1 ‐ Mild disability	133 (27.5)	125 (30.5)	132 (37.9)	99 (33.6)	78 (34.5)	50 (29.6)	34 (30.4)	10 (30.3)
2 ‐ Moderate disability	152 (31.5)	129 (31.5)	84 (24.1)	81 (27.5)	58 (25.7)	41 (24.3)	25 (22.3)	8 (24.2)
3 ‐ Severe disability	101 (20.9)	51 (12.4)	42 (12.1)	33 (11.2)	27 (11.9)	17 (10.1)	17 (15.2)	5 (15.2)
OR (95% CI)		2.0 (1.5, 2.6)	2.6 (1.9, 3.5)	2.4 (1.7, 3.3)	2.7 (1.9, 3.9)	3.6 (2.4, 5.4)	2.3 (1.4, 3.6)	2.6 (1.2, 5.7)
*F* Value: 8.7; *P* < .0001								
Limb Posture, *N* (%)								
0 ‐ No disability	43 (8.9)	46 (11.2)	32 (9.2)	30 (10.2)	23 (10.2)	23 (13.7)	15 (13.4)	6 (18.2)
1 ‐ Mild disability	75 (15.5)	113 (27.6)	106 (30.5)	86 (29.2)	73 (32.3)	58 (34.5)	40 (35.7)	11 (33.3)
2 ‐ Moderate disability	226 (46.8)	180 (43.9)	149 (42.8)	138 (46.8)	97 (42.9)	68 (40.5)	45 (40.2)	13 (39.4)
3 ‐ Severe disability	139 (28.8)	71 (17.3)	61 (17.5)	41 (13.9)	33 (14.6)	19 (11.3)	12 (10.7)	3 (9.1)
OR (95% CI)		2.7 (2.0, 3.6)	2.6 (1.9, 3.6)	3.1 (2.3, 4.3)	3.5 (2.4, 5.0)	4.8 (3.2, 7.2)	4.7 (2.9, 7.4)	6.3 (2.9, 13.7)
*F* Value: 14.3; *P* < .0001								
Pain, *N* (%)								
0 ‐ No disability	151 (31.3)	170 (41.5)	164 (47.1)	143 (48.5)	109 (48.2)	84 (49.7)	57 (50.9)	18 (54.5)
1 ‐ Mild disability	142 (29.4)	142 (34.6)	111 (31.9)	92 (31.2)	74 (32.7)	52 (30.8)	35 (31.3)	9 (27.3)
2 ‐ Moderate disability	124 (25.7)	74 (18.0)	57 (16.4)	43 (14.6)	35 (15.5)	26 (15.4)	16 (14.3)	5 (15.2)
3 ‐ Severe disability	66 (13.7)	24 (5.9)	16 (4.6)	17 (5.8)	8 (3.5)	7 (4.1)	4 (3.6)	1 (3.0)
OR (95% CI)		2.9 (2.1, 3.9)	3.5 (2.6, 4.9)	3.9 (2.7, 5.5)	4.3 (2.9, 6.3)	4.3 (2.8, 6.6)	4.2 (2.5, 6.9)	5.0 (2.1, 11.8)
*F* Value: 15.1; *P* < .0001								

CI = confidence interval; *N* = number of patients; OR = odds ratio; Tx = treatment session.

* DAS objectively evaluates functional impairment resulting from spasticity across several subscales, including dressing, hygiene, limb posture, and pain.^35^ Patients were scored on a 4‐point scale (range: 0‐3) for each subscale, where a “0” represents no disability and a “3” represents severe disability (normal activities limited). DAS was assessed by the clinician at treatment session 1 and at each subsequent treatment session.

‡ Data were analyzed using ordinal logistic regression. The *F* value and level of significance (*P* value) are shown for each subscale.

† Data from treatment session 1 were used as the reference for statistical analysis.

#### Numeric Pain Rating Scale (NPRS)

**Figure 5:**
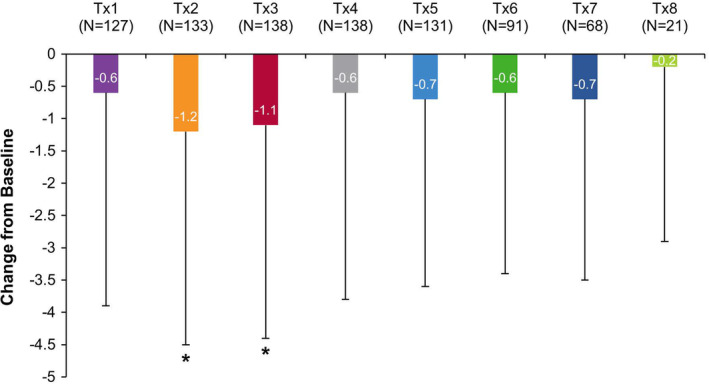
Numeric Pain Rating Scale (NPRS) in the upper limb spasticity population. NPRS is a common scale that is used to assess pain intensity using an 11‐point rating scale (range: 0‐10), where “0” represents no pain and a “10” represents the worst pain imaginable.^36^, ^37^ NPRS was reported by the patient at baseline (prior to treatment) and 5 ± 1 weeks post‐treatment via phone or web. Mean change in NPRS scores vs baseline are shown. *Indicates statistically significant change from baseline at *P* < .006 (Bonferroni correction applied). *N*, number of patients; Tx, treatment session.

The baseline NPRS score (*N* = 463) was 3.8 ± 3.3 (mean ± SD). OnabotulinumtoxinA decreased mean NPRS scores (range: −0.2 to −1.2 points; Figure 5), indicative of reduced patient‐reported spasticity‐related pain, with treatments 2 and 3 statistically significant compared to baseline (both comparisons, *P* < .006).

#### Clinician Satisfaction

**Figure 6:**
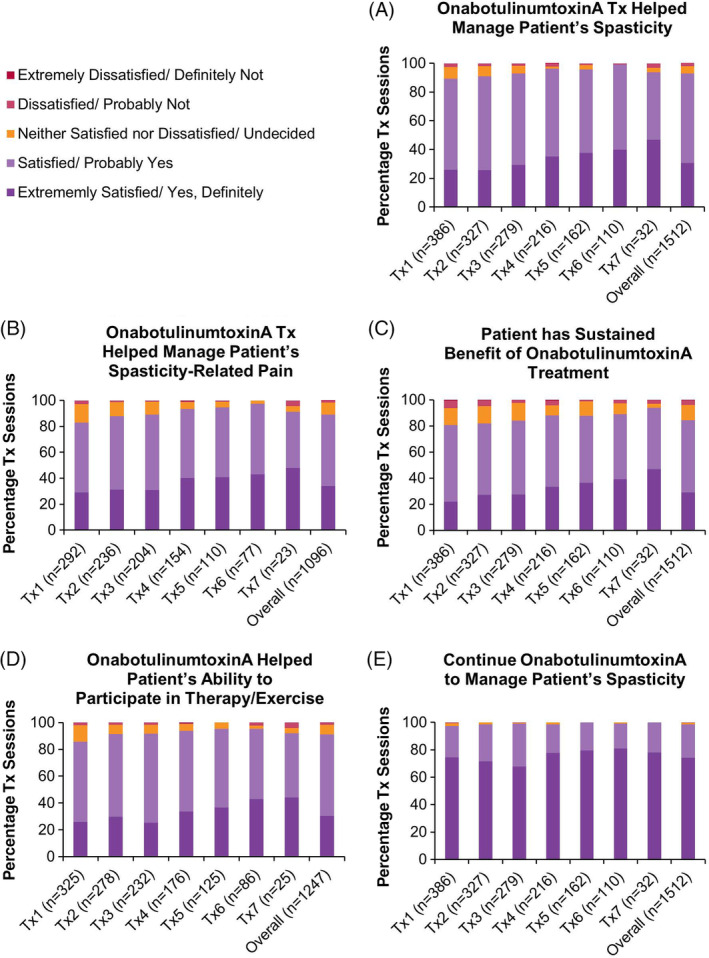
Clinician‐reported satisfaction with onabotulinumtoxinA treatment in the upper limb spasticity population. At the subsequent treatment session, clinicians were asked a series of questions to determine their satisfaction with the previous onabotulinumtoxinA (referred to as BOTOX in the original questionnaire) treatment for spasticity. Therefore, data on clinician satisfaction at the final treatment session and/or treatment session 8 were not gathered. For figures (B) and (D), the percentage of clinicians was recalculated to exclude those that indicated that the question was “not applicable.” Data presented as percentage of treatment sessions. *n*, number of treatment sessions; Tx, treatment session.

Clinicians reported extreme satisfaction/satisfaction that onabotulinumtoxinA helped manage a patient's spasticity (overall: 92.9% of treatment sessions; Figure 6A) and had sustained benefit of treatment (overall: 84.3%; Figure 6C). Additionally, clinicians reported extreme satisfaction/satisfaction that onabotulinumtoxinA helped manage their patient's spasticity‐related pain (overall: 89.0%; Figure 6B) and helped patients participate in therapy/exercise (overall: 91.0%; Figure 6D). Clinicians responded that they would definitely/probably continue onabotulinumtoxinA to manage their patient's spasticity (overall: 98.6%; Figure 6E).

#### Patient Satisfaction

**Figure 7:**
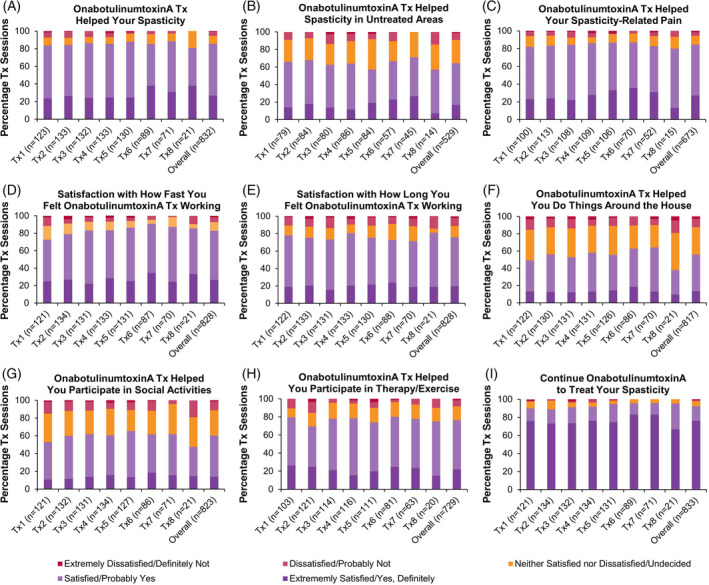
Patient‐reported satisfaction with onabotulinumtoxinA treatment in the upper limb spasticity population. Patients were asked a series of questions to determine their satisfaction with onabotulinumtoxinA (referred to as BOTOX in the original questionnaire) treatment for spasticity 5 ± 1 weeks post‐treatment via phone or web. For figures (B), (C), and (H), the percentage of patients was recalculated to exclude those that indicated that the question was “not applicable.” Data presented as percentage of treatment sessions. *n*, number of treatment sessions; Tx, treatment session.

Patients reported extreme satisfaction/satisfaction that onabotulinumtoxinA helped their spasticity (overall: 85.7% of treatment sessions; Figure 7A) and spasticity‐related pain (overall: 84.5%; Figure 7C), as well as helped them participate in therapy/exercise (overall: 76.4%; Figure 7H). Patients were extremely satisfied/satisfied with how fast (overall: 82.8%; Figure 7D) and how long (overall: 75.7%; Figure 7E) they felt onabotulinumtoxinA working. Patients responded that they would definitely/probably continue onabotulinumtoxinA to manage their spasticity (overall: 92.2%; Figure 7I).

### *Safety and Tolerability*

In total, 563 AEs were reported in 179 patients (37.0%; Table S2), with 15 events in 14 patients (2.9%) considered treatment‐related (Table 4). The most commonly reported treatment‐related AE was muscular weakness, with seven events reported in seven patients (1.4%). In total, 137 serious AEs were reported by 69 patients (14.3%; Table S2). Of these serious AEs, three events in two patients (0.4%) were considered treatment‐related (Table 4): one post‐stroke man experienced muscular weakness of the thumb and a second post‐stroke man experienced dysphagia and slow speech. Neither case was considered related to the distant spread of toxin as adjudicated by a panel of safety clinicians. Both patients experienced the AE following their third onabotulinumtoxinA treatment and returned for several subsequent treatments. An AE leading to withdrawal from the study occurred in five patients; two of these were considered treatment‐related and included muscular weakness and drug tolerance. During the study period, 13 deaths were reported, with 9 of these deaths in the upper limb population; none were considered treatment‐related.

**Table 4:** Treatment‐related adverse events and treatment‐related serious adverse events in the upper limb spasticity population*

	Patients, *N* (%)	Events, *n*
TRAEs		
Muscular weakness	7 (1.4)	7
Dysphagia	2 (0.4)	2
Asthenia	1 (0.2)	1
Drug tolerance	1 (0.2)	1
Gait disturbance	1 (0.2)	1
Grip strength decreased	1 (0.2)	1
Peripheral edema	1 (0.2)	1
Slow speech	1 (0.2)	1
TRSAEs		
Dysphagia	1 (0.2)	1
Muscular weakness	1 (0.2)	1
Slow speech	1 (0.2)	1

*n* = number of adverse events; *N* = number of patients; TRAE = treatment‐related adverse events; TRSAE = treatment‐related serious adverse events.

* All TRAE and TRSAE data are shown.

## Discussion

Controlled trials on the use of onabotulinumtoxinA to treat upper limb post‐stroke spasticity have been published (eg,^23^, ^26^, ^27^, ^39^). However, observational, real‐world data are limited but are recommended to improve patient outcomes. The goals of ASPIRE were to examine onabotulinumtoxinA treatment patterns in a real‐world clinical setting, as well as the effectiveness of onabotulinumtoxinA to treat spasticity using clinician‐ and patient‐reported outcomes. This analysis describes the upper limb spasticity population from ASPIRE, which included patients who received at least one treatment with onabotulinumtoxinA to the upper limb during the 2‐year study.

In ASPIRE, clenched fist was the most frequently treated upper limb clinical presentation, followed by flexed elbow, flexed wrist, pronated forearm, adducted/internally rotated shoulder, thumb‐in‐palm, and intrinsic plus hand. Specific aspects of onabotulinumtoxinA treatment utilization were comparable across clinical presentations. For example, EMG was the most common injection localization method utilized by clinicians, ranging from 57%‐65% of treatment sessions. Additionally, regardless of presentation, ultrasound was used in ~25% of treatment sessions. The use of localization techniques, such as EMG, electrical stimulation (E‐stim), and ultrasound, are recommended to help identify target muscles for injection,^31^, ^40^, ^41^ resulting in increased accuracy of injection placement compared to manual needle placement alone.^42^ Of note, more than one localization method could be recorded per treatment session and localization type may have been influenced by availability of equipment at the study site.

Not surprisingly, onabotulinumtoxinA treatment utilization, such as number of injections, dilution, and dosing, varied by clinical presentation. The highest mean dose of onabotulinumtoxinA, as well as the largest dose range (minimum to maximum = 585 U), was for the treatment of flexed elbow. The maximum dose of onabotulinumtoxinA per presentation was higher than the 400 U recommended by the product label.^19^ Dosing decisions can be influenced by many factors, such as treatment history, patient condition, severity of spasticity, number and location of muscles targeted, AE risk, and experience of clinician.^29^, ^31^, ^43^, ^44^ Indeed, when clinicians were asked about changes to their approach compared to the previous treatment, ~50% of clinicians changed the muscles targeted to better control spasticity and ~39% of clinicians adjusted the dose of onabotulinumtoxinA due to not enough effect in previous muscles treated. A recent Delphi panel agreed with this strategy, recommending an increase in the number of muscles targeted and/or changes to the dose of onabotulinumtoxinA to improve treatment outcomes.^31^ These adjustments likely require instrumented localization techniques, such as those utilized in ASPIRE (ie, EMG and ultrasound), which is in contrast to older studies that did not utilize these methods.^27^ Despite variability in onabotulinumtoxinA utilization, ASPIRE data are consistent with clinical recommendations^6^, ^17^, ^31^, ^43^ and muscles targeted in the upper limb were comparable to previous publications.^15^, ^31^, ^45^, ^46^ Combined, these data reflect the similarities and differences in clinical approaches to treat common spasticity presentations of the upper limb. ASPIRE data speak to the individualized nature of onabotulinumtoxinA treatment for spasticity and the need to continually reassess a patient's condition to achieve treatment goals.

As utilization patterns and treatment goals vary, it can be difficult to assess the efficacy of onabotulinumtoxinA for the treatment of spasticity. Clinician‐ and patient‐reported outcomes,^47^, ^48^ such as treatment satisfaction, functional impairment (clinician‐reported DAS^35^), and pain intensity (patient‐reported NPRS^36^, ^37^), can better capture information regarding patient health, quality of life, and functional goals compared to traditional assessments. Data from ASPIRE demonstrate that most clinicians and patients were satisfied with onabotulinumtoxinA treatment throughout the study and would continue to use onabotulinumtoxinA to manage spasticity. In support of these findings, only 9.3% of upper limb spasticity patients discontinued ASPIRE due to ineffective treatment. In alignment with recommendations on combining onabotulinumtoxinA treatment with rehabilitation,^18^, ^22^, ^49^ most clinicians and patients in ASPIRE reported extreme satisfaction/satisfaction that treatment helped patients participate in therapy/exercise. Similarly, recurrent onabotulinumtoxinA significantly reduced DAS scores (dressing, hygiene, limb posture, and pain), demonstrating improved quality of life with repeated, long‐term treatment. Increased disability on the DAS has been associated with diminished health‐related quality of life in post‐stroke spasticity patients.^50^ Therefore, a reduction in DAS scores following onabotulinumtoxinA treatment in ASPIRE demonstrates a meaningful outcome for patients. Lastly, similar to previous studies,^51^, ^52^, ^53^, ^54^, ^55^, ^56^, ^57^ onabotulinumtoxinA treatment significantly reduced patient‐reported spasticity‐related pain. This is an important finding from ASPIRE, as spasticity‐related pain has been associated with reduced quality of life^58^, ^59^ and decreased work productivity, leading to economic losses.^60^

The safety and tolerability of onabotulinumtoxinA to treat post‐stroke spasticity (pooled analysis^61^) and other etiologies (reviewed in^62^, ^63^, ^64^) has been demonstrated previously. Safety and tolerability data from large observational studies, such as ASPIRE, have generalizability to clinical settings and help to demonstrate the complexity of treating spasticity patients with onabotulinumtoxinA in the real‐world. Data from ASPIRE support the safety of onabotulinumtoxinA to treat adult spasticity across a wide range of doses, from multiple etiologies and geographical regions, and in patients naïve or non‐naïve to botulinum toxins for spasticity. OnabotulinumtoxinA demonstrated an acceptable safety profile, with no new safety signals identified. Adverse event data captured in ASPIRE are consistent with safety data within the literature and the package insert.^19^, ^23^, ^26^, ^27^

One strength of the ASPIRE study is its size. ASPIRE is the largest known adult spasticity registry, with data from 54 international sites, across 7 countries, including the United States, Spain, Germany, the United Kingdom, France, Italy, and Taiwan. In addition, ASPIRE data were gathered across a range of underlying etiologies of spasticity, including stroke, multiple sclerosis, cerebral palsy, traumatic brain injury, and spinal cord injury. ASPIRE data represent real‐world clinical practice and reflect the similarities and differences in spasticity management approaches by clinicians. For these reasons, data from ASPIRE has increased external validity and generalizability and provides information incremental to published controlled trials.

As discussed previously,^32^ there are limitations to large, observational registries, like ASPIRE. As data were collected in the real‐world, there was a lack of control over study elements and confounding factors. The length of the study may have contributed to patient dropout, leading to diminishing sample size at later treatment sessions and the need for caution when interpreting these results. Similarly, PRO data gathered outside of the clinic via phone or web, such as NPRS or patient satisfaction, also showed diminishing sample size, which may indicate that patients found these assessments burdensome to complete. As patients discontinued the study, reported outcomes, such as satisfaction or DAS, at later timepoints may disproportionally represent those that were satisfied with, and/or responsive to, treatment. The reasons why clinicians chose onabotulinumtoxinA vs another toxin were not gathered; however, patient characteristics could have influenced a clinician's choice to use onabotulinumtoxinA, thus affecting ASPIRE patient demographics. As post‐stroke patients were the largest population in the study, ASPIRE data are highly reflective of this etiology and may have more limited applicability to other etiologies. The upper limb population included patients that received at least one treatment with onabotulinumtoxinA to the upper limb but may have also received treatment to the lower limb. Therefore, data representative of more holistic improvements following onabotulinumtoxinA treatment (eg, PROs), may not be exclusive to the upper limb. Inclusion of non‐naïve patients may have biased the patient population in favor of those in which onabotulinumtoxinA was tolerable and effective; however, inclusion of both naïve and non‐naïve patients in this analysis is reflective of the varied treatment history observed in real‐world clinical practice. Future analyses from ASPIRE will need to investigate factors, such as treatment history or regional differences, to better understand how they influence onabotulinumtoxinA utilization and outcomes to further guide spasticity management strategies to improve patient care.

## Conclusions

ASPIRE provides valuable, real‐world data on onabotulinumtoxinA utilization for the treatment of adult upper limb spasticity over a 2‐year period across a range of underlying etiologies and geographical regions. ASPIRE captured the individualized nature of onabotulinumtoxinA utilization for upper limb spasticity, with consistently high clinician‐ and patient‐reported satisfaction. Data from this primary analysis, combined with controlled trial data, will guide clinical use of onabotulinumtoxinA, as well as provide insights to improve educational programs on spasticity management.

### *Data Sharing Statement*

Data reported are available within the article and its supplementary materials. Allergan will share deidentified patient‐level data and/or study‐level data, including protocols and clinical study reports, for phase 2 to 4 trials completed after 2008 that are registered on ClinicalTrials.gov or EudraCT. The indication studied in the trial must have regulatory approval in the United States and/or the European Union and the primary manuscript from the trial must be published prior to data sharing. To request access, the researcher must sign a data use agreement. All shared data are to be used for noncommercial purposes only. More information can be found at http://www.allerganclinicaltrials.com.

## Supporting information


**Table S1**

## References

[1] Pandyan AD , Gregoric M , Barnes MP , et al. Spasticity: clinical perceptions, neurological realities and meaningful measurement. Disabil Rehabil. 2005;27(1–2):2‐6. 15799140 10.1080/09638280400014576

[2] Tardieu G , Shentoub S , Delarue R . Research on a technic for measurement of spasticity. Rev Neurol (Paris). 1954;91(2):143‐144. 14358132

[3] Dressler D , Bhidayasiri R , Bohlega S , et al. Defining spasticity: a new approach considering current movement disorders terminology and botulinum toxin therapy. J Neurol. 2018;265(4):856‐862. 29423615 10.1007/s00415-018-8759-1

[4] Kheder A , Nair KP . Spasticity: pathophysiology, evaluation and management. Pract Neurol. 2012;12(5):289‐298. 22976059 10.1136/practneurol-2011-000155

[5] Nair KP , Marsden J . The management of spasticity in adults. BMJ. 2014;349:g4737. 25096594 10.1136/bmj.g4737

[6] Sheean G , Lannin NA , Turner‐Stokes L , Rawicki B , Snow BJ . Botulinum toxin assessment, intervention and after‐care for upper limb hypertonicity in adults: international consensus statement. Eur J Neurol. 2010;17(Suppl 2):74‐93. 20633180 10.1111/j.1468-1331.2010.03129.x

[7] Trompetto C , Marinelli L , Mori L , et al. Pathophysiology of spasticity: implications for neurorehabilitation. Biomed Res Int. 2014;2014:354906. 25530960 10.1155/2014/354906 PMC4229996

[8] Esquenazi A , Albanese A , Chancellor MB , et al. Evidence‐based review and assessment of botulinum neurotoxin for the treatment of adult spasticity in the upper motor neuron syndrome. Toxicon. 2013;67:115‐128. 23220492 10.1016/j.toxicon.2012.11.025

[9] Esquenazi A , Mayer NH . Instrumented assessment of muscle overactivity and spasticity with dynamic polyelectromyographic and motion analysis for treatment planning. Am J Phys Med Rehabil. 2004;83(10 Suppl):S19‐S29. 15448574 10.1097/01.phm.0000141127.63160.3e

[10] Svensson J , Borg S , Nilsson P . Costs and quality of life in multiple sclerosis patients with spasticity. Acta Neurol Scand. 2014;129(1):13‐20. 23683163 10.1111/ane.12139

[11] Pattuwage L , Olver J , Martin C , et al. Management of spasticity in moderate and severe traumatic brain injury: evaluation of clinical practice guidelines. J Head Trauma Rehabil. 2017;32(2):E1‐E12. 10.1097/HTR.0000000000000234 27120291

[12] Ganapathy V , Graham GD , DiBonaventura MD , Gillard PJ , Goren A , Zorowitz RD . Caregiver burden, productivity loss, and indirect costs associated with caring for patients with poststroke spasticity. Clin Interv Aging. 2015;10:1793‐1802. 26609225 10.2147/CIA.S91123 PMC4644168

[13] Lundstrom E , Smits A , Borg J , Terent A . Four‐fold increase in direct costs of stroke survivors with spasticity compared with stroke survivors without spasticity: the first year after the event. Stroke. 2010;41(2):319‐324. 20044535 10.1161/STROKEAHA.109.558619

[14] Sturm JW , Donnan GA , Dewey HM , et al. Quality of life after stroke: the north East Melbourne stroke incidence study (NEMESIS). Stroke. 2004;35(10):2340‐2345. 15331799 10.1161/01.STR.0000141977.18520.3b

[15] Thibaut A , Chatelle C , Ziegler E , Bruno MA , Laureys S , Gosseries O . Spasticity after stroke: physiology, assessment and treatment. Brain Inj. 2013;27(10):1093‐1105. 23885710 10.3109/02699052.2013.804202

[16] Ward AB . Spasticity treatment with botulinum toxins. J Neural Transm. 2008;115(4):607‐616. 18389166 10.1007/s00702-007-0833-2

[17] Physicians RCo. Spasticity in adults . Management Using Botulinum Toxin. 2nd ed. London: Royal College of Physicians, Cambrian Typesetters; 2018.

[18] Mills PB , Finlayson H , Sudol M , O'Connor R . Systematic review of adjunct therapies to improve outcomes following botulinum toxin injection for treatment of limb spasticity. Clin Rehabil. 2016;30(6):537‐548. 26198891 10.1177/0269215515593783

[19] Allergan . BOTOX® [Package Insert]. https://media.allergan.com/actavis/actavis/media/allergan‐pdf‐documents/product‐prescribing/20190620‐BOTOX‐100‐and‐200‐Units‐v3‐0USPI1145‐v2‐0MG1145.pdf. Accessed December 17, 2018.

[20] Burstein R , Zhang X , Levy D , Aoki KR , Brin MF . Selective inhibition of meningeal nociceptors by botulinum neurotoxin type a: therapeutic implications for migraine and other pains. Cephalalgia. 2014;34(11):853‐869. 24694964 10.1177/0333102414527648 PMC4167963

[21] Patel AT . Successful treatment of long‐term, poststroke, upper‐limb spasticity with onabotulinumtoxinA. Phys Ther. 2011;91(11):1636‐1641. 21868610 10.2522/ptj.20100370

[22] Giovannelli M , Borriello G , Castri P , Prosperini L , Pozzilli C . Early physiotherapy after injection of botulinum toxin increases the beneficial effects on spasticity in patients with multiple sclerosis. Clin Rehabil. 2007;21(4):331‐337. 17613573 10.1177/0269215507072772

[23] Kaji R , Osako Y , Suyama K , et al. Botulinum toxin type a in post‐stroke upper limb spasticity. Curr Med Res Opin. 2010;26(8):1983‐1992. 20569068 10.1185/03007995.2010.497103

[24] Simpson DM , Gracies JM , Yablon SA , Barbano R , Brashear A . Botulinum neurotoxin versus tizanidine in upper limb spasticity: a placebo‐controlled study. J Neurol Neurosurg Psychiatry. 2009;80(4):380‐385. 18977811 10.1136/jnnp.2008.159657

[25] Jahangir AW , Tan HJ , Norlinah MI , et al. Intramuscular injection of botulinum toxin for the treatment of wrist and finger spasticity after stroke. Med J Malaysia. 2007;62(4):319‐322. 18551937

[26] Childers MK , Brashear A , Jozefczyk P , et al. Dose‐dependent response to intramuscular botulinum toxin type a for upper‐limb spasticity in patients after a stroke. Arch Phys Med Rehabil. 2004;85(7):1063‐1069. 15241751 10.1016/j.apmr.2003.10.015

[27] Brashear A , Gordon MF , Elovic E , et al. Intramuscular injection of botulinum toxin for the treatment of wrist and finger spasticity after a stroke. N Engl J Med. 2002;347(6):395‐400. 12167681 10.1056/NEJMoa011892

[28] Marciniak CM , Harvey RL , Gagnon CM , et al. Does botulinum toxin type a decrease pain and lessen disability in hemiplegic survivors of stroke with shoulder pain and spasticity?: a randomized, double‐blind, placebo‐controlled trial. Am J Phys Med Rehabil. 2012;91(12):1007‐1019. 23064478 10.1097/PHM.0b013e31826ecb02

[29] Pathak MS , Nguyen HT , Graham HK , Moore AP . Management of spasticity in adults: practical application of botulinum toxin. Eur J Neurol. 2006;13(Suppl 1):42‐50. 16417597 10.1111/j.1468-1331.2006.01444.x

[30] Esquenazi A , Novak I , Sheean G , Singer BJ , Ward AB . International consensus statement for the use of botulinum toxin treatment in adults and children with neurological impairments–introduction. Eur J Neurol. 2010;17(Suppl 2):1‐8. 10.1111/j.1468-1331.2010.03125.x 20633176

[31] Simpson DM , Patel AT , Alfaro A , et al. OnabotulinumtoxinA injection for poststroke upper‐limb spasticity: guidance for early injectors from a Delphi panel process. PM R. 2017;9(2):136‐148. 27346090 10.1016/j.pmrj.2016.06.016

[32] Francisco GE , Bandari DS , Bavikatte G , et al. Adult spasticity international registry study: methodology and baseline patient, healthcare provider, and caregiver characteristics. J Rehabil Med. 2017;49(8):659‐666. 28805237 10.2340/16501977-2245

[33] Allergan . BOTOX® 100 Units Summary of Product Characteristics (SmPC). 2018 https://www.medicines.org.uk/emc/product/859/smpc. Accessed December 17, 2018.

[34] Abolhasani H , Ansari NN , Naghdi S , Mansouri K , Ghotbi N , Hasson S . Comparing the validity of the Modified Modified Ashworth Scale (MMAS) and the Modified Tardieu Scale (MTS) in the assessment of wrist flexor spasticity in patients with stroke: protocol for a neurophysiological study. BMJ Open. 2012;2(6):e001394. 10.1136/bmjopen-2012-001394 PMC3532966 23166123

[35] Brashear A , Zafonte R , Corcoran M , et al. Inter‐ and intrarater reliability of the Ashworth scale and the disability assessment scale in patients with upper‐limb poststroke spasticity. Arch Phys Med Rehabil. 2002;83(10):1349‐1354. 12370866 10.1053/apmr.2002.35474

[36] Farrar JT , Polomano RC , Berlin JA , Strom BL . A comparison of change in the 0‐10 numeric rating scale to a pain relief scale and global medication performance scale in a short‐term clinical trial of breakthrough pain intensity. Anesthesiology. 2010;112(6):1464‐1472. 20463579 10.1097/ALN.0b013e3181de0e6d PMC2884191

[37] Farrar JT , Young JP Jr , LaMoreaux L , Werth JL , Poole RM . Clinical importance of changes in chronic pain intensity measured on an 11‐point numerical pain rating scale. Pain. 2001;94(2):149‐158. 11690728 10.1016/S0304-3959(01)00349-9

[38] Mayer NH , Esquenazi A . Muscle overactivity and movement dysfunction in the upper motoneuron syndrome. Phys Med Rehabil Clin N Am. 2003;14(4):855‐883, vii‐viii. 14580042 10.1016/s1047-9651(03)00093-7

[39] Elovic EP , Brashear A , Kaelin D , et al. Repeated treatments with botulinum toxin type a produce sustained decreases in the limitations associated with focal upper‐limb poststroke spasticity for caregivers and patients. Arch Phys Med Rehabil. 2008;89(5):799‐806. 18452724 10.1016/j.apmr.2008.01.007

[40] Childers MK . The importance of electromyographic guidance and electrical stimulation for injection of botulinum toxin. Phys Med Rehabil Clin N Am. 2003;14(4):781‐792. 14580037 10.1016/s1047-9651(03)00047-0

[41] Walker HW , Lee MY , Bahroo LB , Hedera P , Charles D . Botulinum toxin injection techniques for the management of adult spasticity. PM R. 2015;7(4):417‐427. 25305369 10.1016/j.pmrj.2014.09.021

[42] Chan AK , Finlayson H , Mills PB . Does the method of botulinum neurotoxin injection for limb spasticity affect outcomes? A systematic review. Clin Rehabil. 2017;31(6):713‐721. 27370102 10.1177/0269215516655589

[43] Brin MF . Dosing, administration, and a treatment algorithm for use of botulinum toxin a for adult‐onset spasticity. Spasticity Study Group Muscle Nerve Suppl. 1997;6:S208‐S220. 10.1002/(sici)1097-4598(1997)6+<208::aid-mus15>3.0.co;2-1 9826992

[44] Sunnerhagen KS , Olver J , Francisco GE . Assessing and treating functional impairment in poststroke spasticity. Neurology. 2013;80(3 Suppl 2):S35‐S44. 23319484 10.1212/WNL.0b013e3182764aa2

[45] Nalysnyk L , Papapetropoulos S , Rotella P , Simeone JC , Alter KE , Esquenazi A . OnabotulinumtoxinA muscle injection patterns in adult spasticity: a systematic literature review. BMC Neurol. 2013;13:118. 24011236 10.1186/1471-2377-13-118 PMC3848723

[46] Mayer NH , Esquenazi A , Childers MK . Common patterns of clinical motor dysfunction. Muscle Nerve Suppl. 1997;6:S21‐S35. 9826981

[47] Deshpande PR , Rajan S , Sudeepthi BL , Abdul Nazir CP . Patient‐reported outcomes: a new era in clinical research. Perspect Clin Res. 2011;2(4):137‐144. 22145124 10.4103/2229-3485.86879 PMC3227331

[48] Weldring T , Smith SM . Patient‐reported outcomes (PROs) and patient‐reported outcome measures (PROMs). Health Serv Insights. 2013;6:61‐68. 25114561 10.4137/HSI.S11093 PMC4089835

[49] Rosales RL , Efendy F , Teleg ES , et al. Botulinum toxin as early intervention for spasticity after stroke or non‐progressive brain lesion: a meta‐analysis. J Neurol Sci. 2016;371:6‐14. 27871449 10.1016/j.jns.2016.10.005

[50] Doan QV , Brashear A , Gillard PJ , et al. Relationship between disability and health‐related quality of life and caregiver burden in patients with upper limb poststroke spasticity. PM R. 2012;4(1):4‐10. 22200567 10.1016/j.pmrj.2011.10.001

[51] Wissel J , Muller J , Dressnandt J , et al. Management of spasticity associated pain with botulinum toxin A. J Pain Symptom Manage. 2000;20(1):44‐49. 10946168 10.1016/s0885-3924(00)00146-9

[52] Esquenazi A , Mayer N , Lee S , et al. Patient registry of outcomes in spasticity care. Am J Phys Med Rehabil. 2012;91(9):729‐746. 22469872 10.1097/PHM.0b013e31824fa9ca

[53] Pierson SH , Katz DI , Tarsy D . Botulinum toxin A in the treatment of spasticity: functional implications and patient selection. Arch Phys Med Rehabil. 1996;77(7):717‐721. 8670002 10.1016/s0003-9993(96)90015-5

[54] Bhakta BB , Cozens JA , Bamford JM , Chamberlain MA . Use of botulinum toxin in stroke patients with severe upper limb spasticity. J Neurol Neurosurg Psychiatry. 1996;61(1):30‐35. 8676154 10.1136/jnnp.61.1.30 PMC486452

[55] Marco E , Duarte E , Vila J , et al. Is botulinum toxin type A effective in the treatment of spastic shoulder pain in patients after stroke? A double‐blind randomized clinical trial. J Rehabil Med. 2007;39(6):440‐447. 17624477 10.2340/16501977-0066

[56] Suputtitada A , Suwanwela NC . The lowest effective dose of botulinum A toxin in adult patients with upper limb spasticity. Disabil Rehabil. 2005;27(4):176‐184. 15824048 10.1080/09638280400009360

[57] Baker JA , Pereira G . The efficacy of Botulinum Toxin A for spasticity and pain in adults: a systematic review and meta‐analysis using the grades of recommendation, assessment, development and evaluation approach. Clin Rehabil. 2013;27(12):1084‐1096. 23864518 10.1177/0269215513491274

[58] Andresen SR , Biering‐Sorensen F , Hagen EM , Nielsen JF , Bach FW , Finnerup NB . Pain, spasticity and quality of life in individuals with traumatic spinal cord injury in Denmark. Spinal Cord. 2016;54(11):973‐979. 27067654 10.1038/sc.2016.46

[59] Harrison RA , Field TS . Post stroke pain: identification, assessment, and therapy. Cerebrovasc Dis. 2015;39(3–4):190‐201. 25766121 10.1159/000375397

[60] Lang AM . Botulinum toxin type A therapy in chronic pain disorders. Arch Phys Med Rehabil. 2003;84(3 Suppl 1):S69‐S73; quiz S74‐65. 12708561 10.1053/apmr.2003.50121

[61] Turkel CC , Bowen B , Liu J , Brin MF . Pooled analysis of the safety of botulinum toxin type a in the treatment of poststroke spasticity. Arch Phys Med Rehabil. 2006;87(6):786‐792. 16731213 10.1016/j.apmr.2006.02.015

[62] Tilton AH . Evidence‐based review of safety and efficacy in cerebral palsy. Toxicon. 2015;107(Pt A):105‐108. 26403867 10.1016/j.toxicon.2015.09.020

[63] Naumann M , Jankovic J . Safety of botulinum toxin type A: a systematic review and meta‐analysis. Curr Med Res Opin. 2004;20(7):981‐990. 15265242 10.1185/030079904125003962

[64] Zakin E , Simpson D . Evidence on botulinum toxin in selected disorders. Toxicon. 2018;147:134‐140. 29408357 10.1016/j.toxicon.2018.01.019

