# Supplementary material for: Individualized OnabotulinumtoxinA Treatment for Upper Limb Spasticity Resulted in High Clinician‐ and Patient‐Reported Satisfaction: Long‐Term Observational Results from the ASPIRE Study
Source: PM R. 2020 Feb 27;12(11):1120–33. doi: 10.1002/pmrj.12328 (PMC7687094; doi:10.1002/pmrj.12328)
Supplement: Supplementary file 1 — Table S1 [file PMRJ-12-1120-s001.pdf]

SUPPORTING INFORMATION

Individualized OnabotulinumtoxinA Treatment for Upper Limb Spasticity Resulted in High Clinician- and Patient-Reported Satisfaction: Long-Term Observational Results from the ASPIRE Study

Supplemental Figure 1. Diagram of ASPIRE Study Design and Analysis.

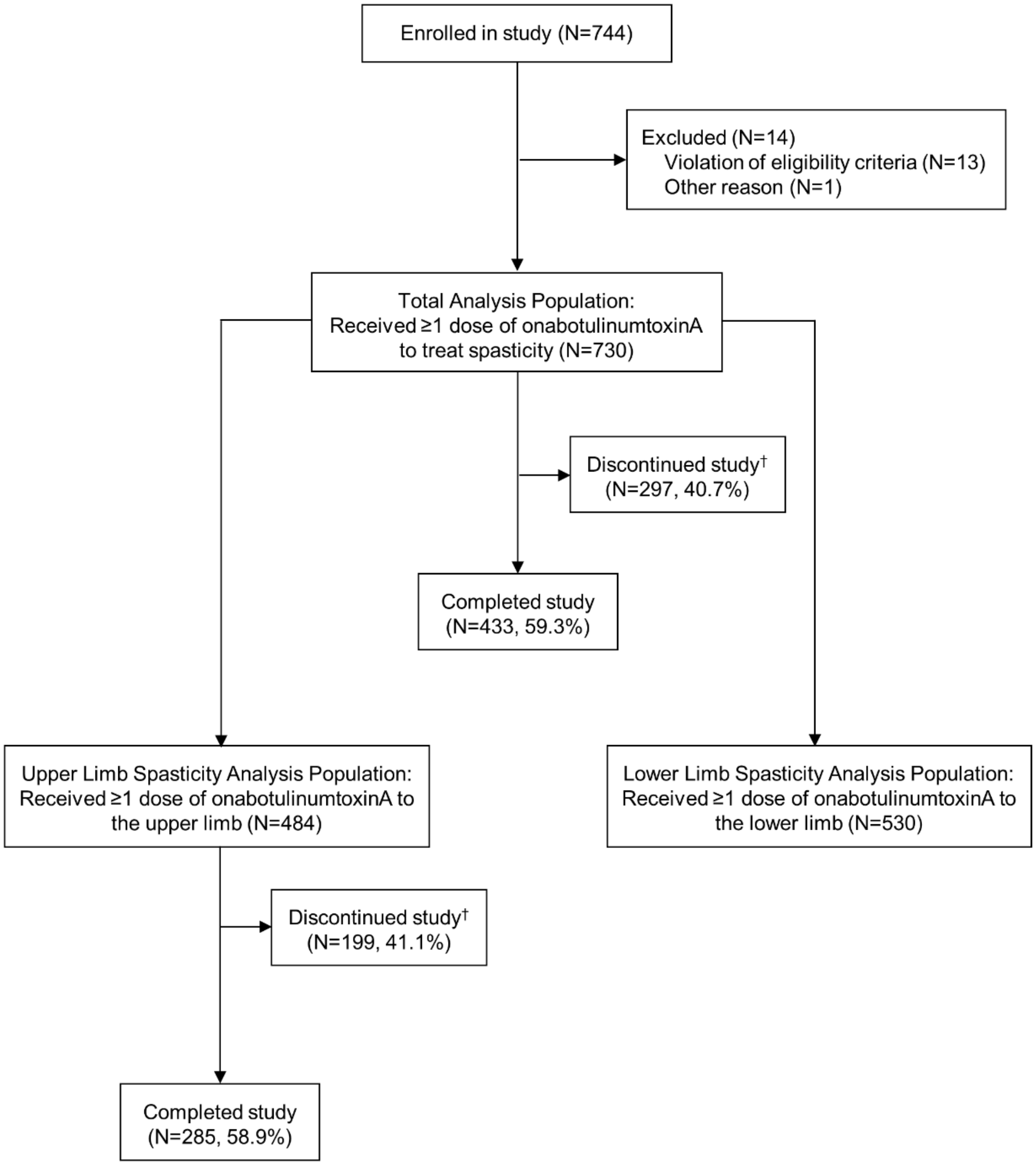

Supplemental Figure 1. Diagram of ASPIRE Study Design and Analysis. †Discontinued study includes all patients who withdrew consent, failed to complete the Final Assessment form, or were lost to follow-up (defined as “unable to contact after 3 attempts”). Reasons for study discontinuation were mutually exclusive. N, number of patients.

Supplemental Table 1. Patient-Reported Reasons for Withdrawal of Consent in the Upper Limb Spasticity Population†

Total (N=484)	N (% of total)
Patient thought the treatment was ineffective	45 (9.3)
Patient had difficulty paying for the onabotulinumtoxinA treatment	25 (5.2)
Related to a change or loss in health care	17 (3.5)
Related to an increase in out-of-pocket expenses	15 (3.1)
Changed to another type of botulinum toxin	15 (3.1)
Changed physicians	9 (1.9)
Patient thought it was inconvenient to come in for treatment visits	9 (1.9)
Changed to another treatment modality	7 (1.4)
Patient thought the injections were too painful	7 (1.4)
Patient moved too far away from treating physician	6 (1.2)
Spasticity improved and no longer needed botulinum toxin treatment	6 (1.2)
Patient experienced transportation difficulties	6 (1.2)
Concerned about risks	3 (0.6)
Physician directed	3 (0.6)
Patient was pregnant, trying to get pregnant, or nursing during the study	1 (0.2)
Side effect or other health problem	1 (0.2)
Other reason‡	26 (5.4)

N, number of patients.

†More than one reason for discontinuation could have been selected; categories are not mutually exclusive.

‡Other reasons most often cited for discontinuation included death of patient, protocol violations, site closure, and administrative issues.

Supplemental Table 2. Adverse Events and Serious Adverse Events in >1% of Patients in the Upper Limb Spasticity Population

	Patients, N (%)	Events, n
AEs		
Fall	25 (5.0)	31
Urinary tract infection	14 (2.9)	19
Pneumonia	11 (2.3)	11
Musculoskeletal pain	10 (2.1)	10
Musculoskeletal weakness	10 (2.1)	12
Upper respiratory tract infection	9 (1.9)	12
Arthralgia	9 (1.9)	11
Constipation	9 (1.9)	10
Seizure	9 (1.9)	9
Depression	9 (1.9)	9
Dizziness	8 (1.7)	10
Back pain	8 (1.7)	9
Hypertension	7 (1.4)	8
Headache	7 (1.4)	8
Bronchitis	7 (1.4)	8
Pain in extremity	7 (1.4)	7
Peripheral edema	6 (1.2)	6
Asthenia	6 (1.2)	6
SAEs		
Pneumonia	9 (1.9)	9

AE, adverse events; n, number of AEs; N, number of patients; SAE, serious AEs.
